# The global prevalence of sexual dysfunction in obese and overweight women: a systematic review and meta-analysis

**DOI:** 10.1186/s12905-023-02544-4

**Published:** 2023-07-15

**Authors:** Nader Salari, Razie Hasheminezhad, Tabassom Sedighi, Hosna Zarei, Shamarina Shohaimi, Masoud Mohammadi

**Affiliations:** 1 Department of Biostatistics, School of Health, Kermanshah University of Medical Sciences, Kermanshah, Iran; 2 Sleep Disorders Research Center, Kermanshah University of Medical Sciences, Kermanshah, Iran; 3 Student Research Committee, Kermanshah University of Medical Sciences, Kermanshah, Iran; 4 Faculty of Health, Education, Medicine and Social Care, School of Medicine, Vision and Eye Research Institute, Anglia Ruskin University, Cambridge, UK; 5 Department of Biology, Faculty of Science, University Putra Malaysia, Serdang, Selangor Malaysia; 6 Cellular and Molecular Research Center, Gerash University of Medical Sciences, Gerash, Iran

**Keywords:** Female Sexual Dysfunction, FSD, Obesity, Overweight

## Abstract

**Background:**

Obesity is a pressing public health risk issue worldwide. Women, in particular, face a higher risk of obesity. Recent research has highlighted the association between obesity and female sexual dysfunction. Therefore, the objective of this study is to investigate the global prevalence of sexual dysfunction in obese and overweight women through a systematic review and meta-analysis.

**Methods:**

In this study, a systematic search was conducted across electronic databases, including PubMed, Scopus, Web of Science, Embase, ScienceDirect, and Google Scholar. The search aimed to identify studies published between December 2000 and August 2022 that reported metabolic syndrome's impact on female sexual dysfunction.

**Results:**

The review included nine studies with a sample size of 1508 obese women. The I^2^ heterogeneity index indicated high heterogeneity (I^2^: 97.5). As a result, the random effects method was used to analyze the data. Based on this meta-analysis, the prevalence of sexual dysfunction in women with obesity was reported as 49.7% (95%CI: 35.8–63.5). Furthermore, the review comprised five studies involving 1411 overweight women. The I^2^ heterogeneity test demonstrated high heterogeneity (I^2^: 96.6). Consequently, the random effects model was used to analyze the results. According to the meta-analysis, the prevalence of sexual dysfunction in overweight women was 26.9% (95% CI: 13.5–46.5).

**Conclusion:**

Based on the results of this study, it has been reported that being overweight and particularly obese is an important factor affecting women's sexual dysfunction. Therefore, health policymakers must acknowledge the significance of this issue in order to raise awareness in society about its detrimental effect on the female population.

## Background

Obesity and overweight refer to the excessive and abnormal accumulation of body fat, which leads to adverse health effects [1]. This condition represents a significant public health concern worldwide [2] and has detrimental health effects on individual well-being and society’s financial burden [3].

The increasing prevalence of high body mass index (BMI) and its associated mortality rates pose a significant threat to people's health in many countries [3]. However, there are distinct factors that contribute to the higher vulnerability of women compared to men in terms of being underweight, overweight, or obese, including variations in biological factors (such as hormones) and behavioural characteristics (such as childhood food deprivation and inadequate physical activity [4].

One possible explanation for this gender disparity is that women tend to experience more difficulties in long-term weight recovery and maintenance as they transition into adulthood and reproductive years [5].

Globally, approximately 40% of women are classified as overweight, while 15% are categorized as obese [6]. In the United States, the prevalence of obesity among adults aged 20–59 years is higher among women (38–41%) compared to men (34–38%) [7].

Epidemiological studies have identified obesity and overweight as significant risk factors for various diseases, including diabetes, certain cancers, cardiovascular diseases, and high blood pressure [3]. Moreover, women who are overweight or obese face a relatively higher risk of experiencing severe maternal complications and mortality [2]. These conditions can also lead to menstrual irregularity, reduced quality and quantity of ovulation, longer time to conceive, and the need for higher doses of medication to stimulate ovulation [8]. Furthermore, women's sexual response cycle is also a complex process influenced by multiple factors, including vascular, nervous, hormonal, and psychogenic factors. Any disruption in these factors can contribute to female sexual dysfunction (FSD) [9]. Additionally, certain treatments for these conditions, such as the use of certain anti-hypertensive drugs or antidepressants, can have negative effects on sexual performance [10].

Sexual dysfunction refers to any condition that hinders a person's ability to derive satisfaction from sexual activity [9]. In particular, female sexual dysfunction (FSD) is a complex disorder with multi-faceted causes rooted in biological and psychosocial factors [11]. FSD can have detrimental effects on an individual’s self-esteem, sense of wholeness, and interpersonal relationships, often leading to emotional discomfort [12].

The symptoms of FSD impact over 40% of adult women worldwide [11]. A comprehensive international clinical study revealed that 39% of sexually active women reported experiencing at least one form of sexual disorder. Notably, during menopause, the prevalence of this disorder ranges between 25 and 79% [13].

The symptoms of FSD impact over 40% of adult women worldwide [11]. A comprehensive international clinical study revealed that 39% of sexually active women reported experiencing at least one form of sexual disorder. Notably, during menopause, the prevalence of this disorder ranges between 25 and 79% [13].

In recent years, research has revealed a correlation between obesity and sexual dysfunction, with a significant number of obese women reporting that their primary sexual dysfunction is related to orgasmic difficulties [14].

Given the numerous complications associated with obesity and overweight and the significance of sexual dysfunction in women, we conducted a comprehensive review of studies in this field. Our objective was to perform a systematic review and meta-analysis to determine the global prevalence of sexual dysfunction among women who are obese or overweight. The findings of this study can provide crucial evidence to shed light on the issue of sexual dysfunction in obese and overweight women worldwide.

## Methods

Our search was conducted from December 2020- August 2022 for this systematic review. Five databases, namely PubMed, Web of Science, Google Scholar, Scopus, ScienceDirect, and Embase, were searched to retrieve relevant articles using the keywords "Female Sexual Dysfunction" and “FSD”. To ensure the comprehensiveness of the search, no restrictions were placed on the publication year of the articles. The gathered information was subsequently organized and managed using the information management software EndNote.

### Study selection

The inclusion criteria for the studies were as follows: cross-sectional studies, studies with accessible full-text versions, and studies that presented adequate data on sample size and prevalence. Conversely, the exclusion criteria encompassed case reports, case series studies, review and duplicate studies.

### Data extraction

**Fig. 1:**
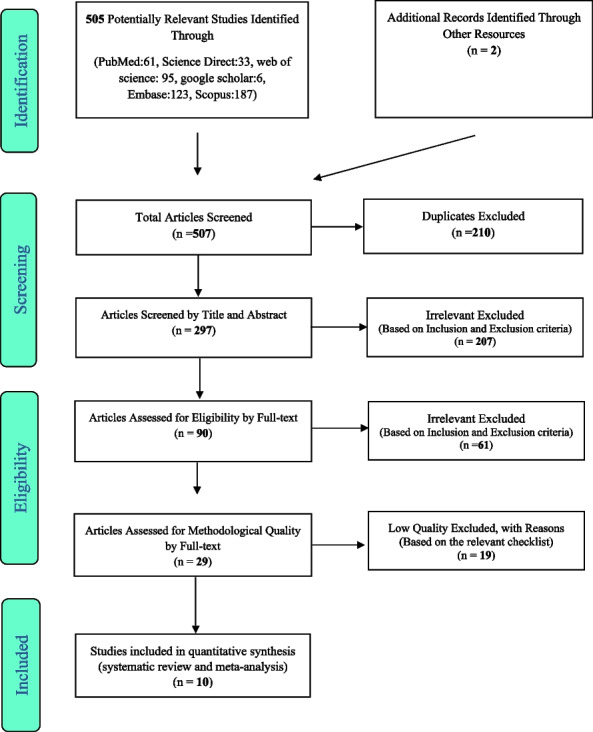
The flowchart on the stages of including the studies in the systematic review and meta-analysis (PRISMA 2009(

The selection of studies followed the guidelines outlined by PRISMA (Preferred Reporting Items for Systematic Reviews and Meta-Analyses). Initially, duplicate studies found across different databases were excluded. To mitigate bias, two researchers independently conducted the review process and data extraction. In total, 505 articles were screened through database searches, and two potentially relevant articles were identified through manual searches. The selected articles were then imported into the information management software EndNote for further analysis. Following the PRISMA steps, the articles underwent thorough evaluation, resulting in the inclusion of 10 studies for the final review. The findings and relevant information from these 10 studies are presented in Table 1 and Fig. 1.

**Table 1:** Summary of characteristics of included studies of the prevalence of sexual dysfunction in obese and overweight women

Author	Year	Location	Age	Type of study	Women sample size	Sample size of obese women	Sample size of overweight women	Prevalence of FSD in obesity and overweight women	Instrument
Shorub et al. [15]	2016	Egypt	30.2 ± 8.7	cross-sectional case–control	107	65	-	FSD in obesity: 81.5%	FSFI
Abu Ali et al. [16]	2009	Jordan	< 50,50–59, ≥ 60	cross-sectional	613	355	195	FSD in obesity: 20.3%, FSD in overweight: 21.2%	FSFI
Karadag et al. [17]	2014	Turkey	18–24	case–control	2081	594	618	FSD in obesity: 14.8%, FSD in overweight:10%	face to face sampling
Yaylali et al. [18]	2010	Turkey	8 ± 36.5	case–control	75	45	-	FSD in obesity:86%,	FSFI
Kadioglu et al. [19]	2009	Turkey	36.0 ∓5.14	case–control	91	64	-	FSD in obesity:50%	FSFI
Mostafa et al. [20]	2017	Egypt	31.2 ∓7.3	cross-sectional	150	17	133	FSD in obesity: 70.6%, FSD in overweight: 34.6%	FSFI, Self-Administered Questionnaire
Silva et al. [21]	2019	Brazil	40–65	cross-sectional	221	68	98	FSD in obesity: 66.2%, FSD in overweight: 57.1%	FSFI
Erenel et al. [22]	2013	Turkey	41.17∓ 8.38	cross-sectional	201	201	-	FSD in obesity: 12.8%	FSFI
Aba et al. [23]	2020	Turkey	18–60	case–control	191	99	-	FSD in obesity: 68.7%	FSFI
Senobari et al. [24]	2019	Iran		cross-sectional	206	-	100	FSD in overweight: 25%	FSFI
			≥ 20

### Quality evaluation and statistical analysis

To validate and assess the quality of articles, a STROBE checklist, designed for observational studies, was utilized. This checklist comprises 32 items. Articles that achieved a score of 16 or higher were categorized as having good or moderate methodological quality, whereas those scoring below 16 were deemed to exhibit poor methodological quality. As a result, articles characterized by poor methodological quality were excluded from the study. The extracted results of the selected studies were entered into Comprehensive Meta-Analysis, version 2 (Biostat Inc, New Jersey). To assess the heterogeneity of the studies, the I^2^ test was employed.

## Results

In this systematic review and meta-analysis, most of the included studies were cross-sectional. Four studies were identified as case–control studies [17–19, 23], and one study was categorized as a case–control-cross-sectional study [15]. Moreover, most of the reviewed studies were conducted in Asian countries, with no studies identified from North America. Among the studies included, nine studies used the standard Female Sexual Function Index (FSFI) questionnaire to assess the presence of sexual dysfunction. One study employed a self-administered version of the FSFI questionnaire [20], while another study used face-to-face interviews [17].

Regarding the studies presented in Table 1, the highest reported prevalence of sexual dysfunction among obese women was 86% in a study conducted by Yaylali et al. in 2010 in Turkey [18]. In overweight women, the highest prevalence was reported as 57.1% in a study conducted by Silva et al. in 2019 in Brazil [21]. Conversely, the lowest prevalence of sexual dysfunction among obese women was reported as 12.8% in a study conducted by Erenel et al. in 2013 in Turkey [22]. For overweight women, the lowest prevalence was reported as 10.0% in a survey conducted by Karadag et al. in 2014 in Turkey [17].

**Fig. 2:**
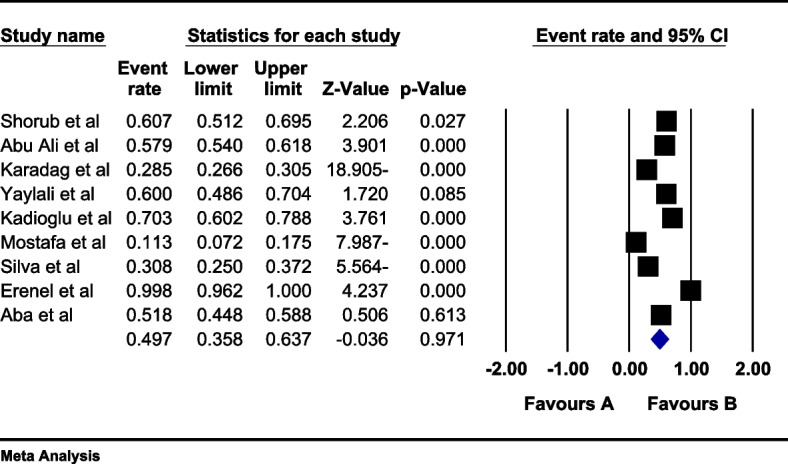
Forest plot of sexual dysfunction in women with obesity based on the random effects model

**Fig. 3:**
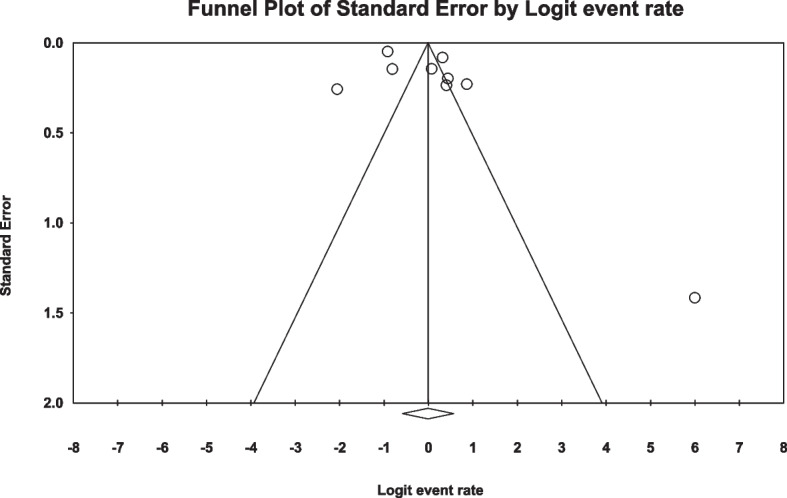
Funnel plot of the publication bias in the reviewed studies

In the review of 9 studies with a total sample size of 1508 obese women, a high level of heterogeneity was observed according to the I^2^ heterogeneity test (I^2^: 97.5). Consequently, the random effects method was employed to analyze the results. Based on the meta-analysis, the prevalence of sexual dysfunction among women with obesity was determined to be 49.7 (95% CI: 35.8–63.5) (Fig. 2). Additionally, a check for publication bias in the included studies was conducted using the Egger, which indicated the absence of publication bias in the studies (*p* = 0.187) (Fig. 3).

**Fig. 4:**
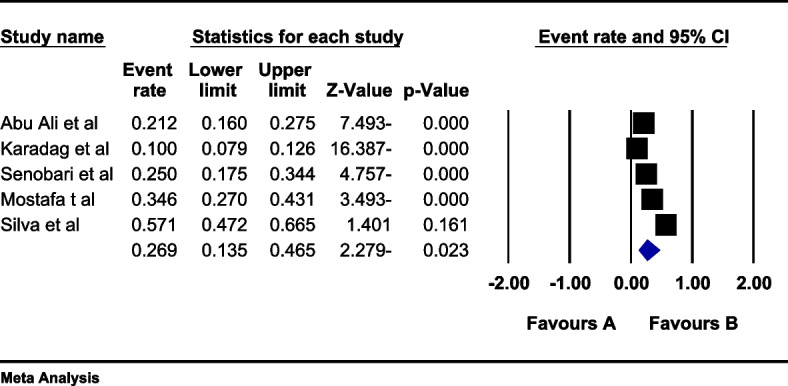
Forest plot of sexual dysfunction in overweight women based on random effects method

**Fig. 5:**
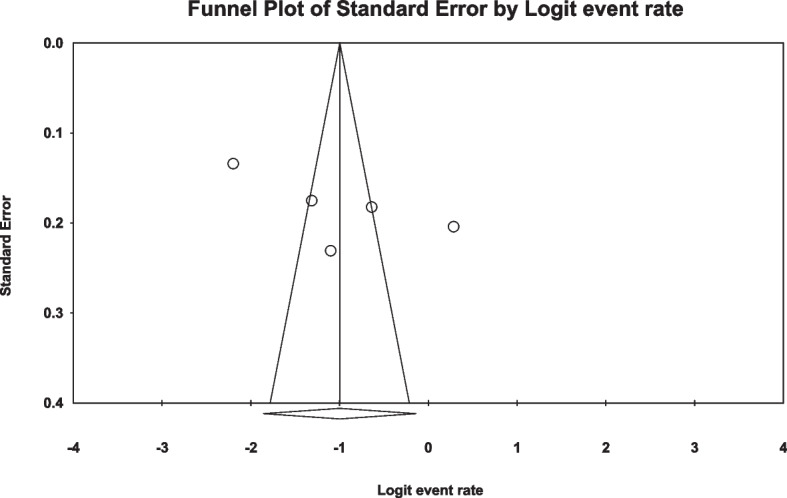
Funnel plot of the publication bias in the reviewed studies

In the review of 5 studies involving a total sample size of 1411 overweight women, the I^2^ heterogeneity test revealed a high-level heterogeneity (I^2^: 96.6). Based on this, the random effects method was used to analyze the results. Based on the meta-analysis, the prevalence of sexual dysfunction among overweight women was 26.9 (95%CI: 13.5–46.5) (Fig. 4). Furthermore, an assessment of publication bias in the included studies was conducted using the Egger test, which indicated the absence of publication bias in the studies (*p* = 0.120) (Fig. 5).

## Discussion

This study represents the first systematic review and meta-analysis focusing on the global prevalence of sexual dysfunction in obese and overweight women. To the best of our knowledge, no previous systematic review study has specifically examined this topic globally. The study employed rigorous secondary analysis methods, selecting and analyzing data from 10 high-quality primary studies.

Given that the prevalence of obesity is higher than that of underweight individuals, except in certain regions of sub-Saharan Africa and Asia, the issue of obesity has emerged as a significant social threat in contemporary times [25]. Female sexual dysfunction (FSD) can affect women of all ages and is often a complex issue with multiple contributing factors [26]. Interestingly, there has been relatively more research focusing on the relationship between obesity and sexual function in men to women, and the impact of obesity on women’s sexual lives remains less understood [27, 28].

Esposito et al. conducted a study comparing women with and without FSD, as well as women with Female Sexual Function Index (FSFI) scores of ≥ 23. Their findings revealed that age and BMI were the only factors associated with FSD [29]. Similarly, Kirchengast et al. (1996), in a study without a control group, reported that body weight and subcutaneous fat, particularly in areas such as the chest, waist, and hips, were associated with a decrease in sexual interest among postmenopausal women who were not undergoing hormone therapy [30]. Notably, these results align with the findings of the present study, further highlighting the association between obesity, overweight status and sexual dysfunction in women.

Several studies have provided insights into the relationship between weight loss and sexual function. It has been observed that lifestyle modifications or drug therapies resulting in weight loss of up to 10% are associated with improvements in sex hormone levels and sexual performance [31–33]. Another study compared the Female Sexual Function Index (FSFI) scores of 52 women with abnormal names? to those of 66 women in the control group. The study found a significant association between FSFI scores and BMI but not the waist-to-age ratio [34]. Similarly, another study found a negative correlation between BMI and female sexual dysfunction. The researcher suggested that individuals with higher BMI might face difficulties with body positioning during sexual activity, emphasizing the importance of early interventions to reduce BMI in healthcare settings [35].

Contrary to our study findings, one study reported no significant relationship between BMI and the improvement of FSFI scores following non-surgical weight loss interventions for obese women. In contrast, non-surgical weight loss in obese men demonstrated more pronounced improvements in sexual dysfunction [14]. Another study found no statistically significant difference in FSFI scores between obese and control groups. The prevalence of female sexual dysfunction was reported to be 50% and 41% in the obese and control groups, respectively [19]. Elofsson et al. also conducted a study involving a Swedish population that included 840 young women (18% overweight, 6% obese) and 426 older women (32% overweight and 11% obese). Their findings indicated no difference in sexual life satisfaction between obese and normal-weight women, contradicting the results of our study [36].

This meta-analysis had certain limitations that should be acknowledged. Firstly, the number of studies available from different countries was unequal, and the participants’ age distributions varied, which could contribute to the differences in the prevalence of obesity and overweight across countries. Additionally, the inclusion of studies was limited to those published in English, potentially leading to the oversight of studies published in other languages. It is important to consider these limitations when interpreting the findings of this meta-analysis.

## Conclusion

The findings of this study reveal the significant prevalence of sexual dysfunction in overweight and obese women. It is crucial to recognize that sexual dysfunction adversely affects the quality of marital relationships and overall life satisfaction for women. Policymakers can use the outcomes of this meta-analysis to emphasize the importance of screening for obesity and overweight among women, as well as raising awareness within society about the detrimental effects of these conditions, including their impact on sexual performance. The results of this research can serve as a valuable research priority and guide interventions aimed at addressing the issue effectively.

## Data Availability

Datasets are available through the corresponding author upon reasonable request.

## Abbreviations

WoS: Web of Science
FSD: Female sexual dysfunction
BMI: Body Mass Index
